# Physiological Psychology

**Published:** 1857-01-01

**Authors:** Robert Dunn


### Art. IX.—PHYSIOLOGICAL PSYCHOLOGY.

No. III.

BY ROBERT DUNN, F.R.C.S. ENG.

*Continued from No. III., p.* 418.

Psychology, the science which investigates *the phenomena* of consciousness, busies itself with *the states, operations, and laws of mind.* Now the human mind—" Illud, *quod sentit, quod sapit, quod vult, quod viget,*"—is " one and indivisible," for the unity of consciousness is the deepest and the most indisputable fact of our nature, and *to feel, to perceive, to think,* and *to will,* are, in truth, so many *acts* or *states* of mind. In other words, intelligence, emotion, and volition, are interwoven with each other, and are *one* at the root. We live in a succession of states, and the fact of the succession of ideas is indisputable. For although, as Sir Henry Holland has well observed, " mental science, from its nature, affords no exact measure of time,—the mind works in a *succession of states.* Two thoughts, or acts of memory, however closely related to one another, cannot be presumed to exist, at *the same instant,* in the consciousness,—each has its own individuality in time. Swiftness of succession naturally suggests unity of time and state, which has no real existence. Nor can the mind maintain two impressions *simultaneously;* and though the succession be uniformly pleasurable or painful, still it is *sequence,* and not coalescence of effects. And thus the ever-changing relation of individual consciousness in the sentient unity, to the different bodily and mental actions, which form the totality of life, illustrates best, though it may not explain, the endless varieties and seeming anomalies of human existence."*

"There exists but *one single principle*," says Dr. Gall, "which sees, feels, tastes, hears, touches, thinks, and wills. But in order that this principle may become capable of perceiving light and sound, of feeling, tasting, touching, and of manifesting the different kinds of thought and propensity, it requires the aid of various material instruments, without which, the exercise of all these faculties would be impossible."†

Still, however, the fact of the *duality* of the brain is not to be questioned. The brain is a double organ, and the symmetrical disposition of the parts of the encephalon on each side of the median plane must be admitted.

But, at the same time, this doubleness of the brain is in harmonious accordance with the doubleness of all the organs of sense; and, indeed, is just what *à priori* reasoning would lead us to expect as necessary to the functions of the special senses, as *double inlets* to knowledge. We have two cerebra, a right and a left brain, or hemisphere; the convolutions, or cerebral organs, are double, and the basement or fundamental ones of the cerebrum—the great internal convolutions, the *ourlet of Foville*—are perfectly symmetrical. The right brain corresponds exactly with the left, just as the right eye or ear corresponds with the left. But it by no means follows from this, as a necessary consequence, and as the late Dr. Wigan‡ has so laboured to prove, that the mind itself is *dual*—in other words, that consciousness is double; and that, because we have two brains, a right and a left brain, so have we *two minds*, each performing its own functions, but in perfect accordance so long as the two brains harmonize in quality, structure, and action with each other. All the information furnished to us by the senses tells of a mind "one and indivisible;" and in every instance in which there is a lateral doubling of the nervous centres, there we find a *commissural band*, like the corpus callosum, the office of which is manifestly that of a bond of union, associating the two sides of the cerebrum in one harmonious action. On this subject, Dr. Todd has well remarked:—

"I can no more infer the existence of two minds, from that of two brains, than I can assume a duality of our visual sense from the existence of two eyes. The two cases, indeed, are strictly analogous. The organic change on each retina developes a corresponding sensorial impression; and from the connexions which subsist between the retinæ,

* *Vide* Sir Henry Holland's Chapters on Mental Physiology. Chap. iii., Mental Consciousness in relation to Time and Succession.

† [illegible]ur les Fonctions de Cerveau, vol. i. p. 243.

‡ *Vide* the Duality of the Mind proved by the Structure, Functions, and Diseases of the Brain, and by the Phenomena of Mental Derangement, and shown to be essential to moral responsibility. By A. L. Wigan, M.D. London, 1844.

and still more from that between the centres of sensation, these impressions become *fused* into *one.* In like manner the organic change in the two brains developing nervous force, in similar modes and proportions, each being capable of affecting the mind similarly, although perhaps not identically, are yet so united in their action, that the double organic affection acts on the mind as *one.* But if, through default of the *connecting media* of the two brains, or through *lesion* of either, the organic changes in each do not harmonize with those of its fellow, then it is plain that two separate and distinct mental affections will result, and that more or less of confusion must ensue. The confusion results from the *want of simultaneous affection of the same mind* by *two separate and distinct brains.* If, in vision, each centre of sensation affected only its own mental phenomena, as Dr. Wigan's theory would compel us to assume, then each mind would perceive a different perspective projection of the object presented to the eyes, and an elaborate and complex mental process would be required to combine the two sensorial impressions. How much simpler is the view of this process, which assigns the combination of the double impression to a physical union in the brain of each physical change in the retina;* so that, in truth, but *one impression*, different from each of its excitant ones, reaches the *mind.* So also in normal intellectual action, the organic changes of the two brains are united by the *various transverse commissures*,† so that but *one* physical stimulus affects the mind, and excites but *one* train of thought. Not so, however, when, from any defect in the brains themselves or in the commissures, the physical conditions necessary for the organic states of the two brains cannot be fulfilled."‡

Still, however, the work of Dr. Wigan "On the Duality of the Mind," is highly suggestive; and, if fairly interpreted by the physiological psychologist, is calculated to throw much light upon *alternating* states of consciousness, delusions, and irregular volitions, as well as upon other obscurities connected with the phenomena of mind.

* Sir D. Brewster has shown that the fact that any near object makes two different perspective projections of itself upon the two retinæ was known so far back as 1613, to the Jesuit Aguilonius, who set himself to inquire how it is that the two dissimilar projections are blended into a *single unconfused image*, and came to the conclusion that it is not by reason of any optical conformity, but by a mental agency which he calls *common sense.* But to Professor Wheatstone belongs the exclusive honour of the *original idea*, and the practical demonstration of its correctness,—that it is on the mental combination of the two dissimilar projections made by a single solid object upon our two retinæ respectively, that our visual perception of its solidity depends; and to this *original* idea we owe his construction of the *Stereoscope.*

† An interesting case, in which the corpus callosum and fornix were imperfect, is published in vol. xxix. of the Royal Medical and Chirurgical Transactions, by Mr. Paget, with some important remarks. It may be fairly inferred that the office of the corpus callosum is that of a band of union to the convoluted surfaces of the hemispheres of the brain, the medium through which the double organic changes in the double cerebral organs are made to correspond with the workings of a single mind.

‡ Dr. Todd's Cyclopædia of Anatomy and Physiology, vol. iii.,—Physiology of the Nervous System, p. 723, *b.*

Sir Henry Holland, in his valuable "Chapters on Mental Physiology," has an admirable essay On the Brain as a Double Organ, showing its compatibility, *as such*, with unity of consciousness. But unity of consciousness does exist, for who can gainsay it? and is therefore, of necessity, compatible with the conformation of the brain as a double organ, however we may argue in explanation of the fact. Nay, more, does not this unity of consciousness, in perception, volition, memory, thought, and passion, constitute the sure and distinguishing characteristic of the sane and healthy mind? But there is another dynamical agency involved in the perceptive consciousness, *volition* or the *will;* and which, though limited in the sphere of its action to consciousness, is a mental element of paramount importance, for it has the power—varying, indeed, in degree in different individuals and at different times in the same individual—of *determining* and *controlling* the *succession* of our states, whether of thought or feeling. In the plenitude of its power it involves the highest attainments of which the human intellect is capable; and this power, be it remembered, of the mind, by the will, to regulate the succession of its states, whether those belonging to perception from *without*, or to thought and memory *within*, varying in different individuals and limited in all, *is given to us not merely to use, but to educate and exalt.* For, it has been well observed, "It is eminently capable of cultivation by steady intention of mind and habitual exercise; and thus, rightly exercised, it becomes one of the highest perfections of our moral and intellectual being. By no quality is one man better distinguished from another, '*than by the power of his will;*' by the mastery acquired over the subject and course of his thoughts; by the power of discarding what is desultory, frivolous, or degrading; and of adhering singly and steadily to those objects which enlarge and invigorate the mind in their pursuit."

"Magni est ingenii revocare mentem a sensibus, et cogitationem a consuetudine revocare."—CICERO, *Tuscul. Quæst.**

And thus, while on the one hand the great and fundamental *mystery of life* consists in the relation of consciousness and volition to the functions of the special senses, and to those of the encephalic organs, which connect man as a sentient, percipient, and intelligent being, with his own organization and with the material world without, the importance, on the other hand, of the duty of sedulously cultivating that dynamical agency which has such a power in determining and controlling the *succession of our ideas*, cannot be overrated, for "the intellectual character of every mental process depends on the manner of suc-

* Sir H. Holland, *Op. ante cit.*

cession, and especially on the action of the will in determining the result.

Now we have seen that *consciousness* with *volition* implies intelligence, and that, as antagonistic to mere consensual and instinctive feeling, it requires the instrumentality of the hemispherical ganglia for its manifestation. For, wherever the essential phenomena are present which formularize the perceptive consciousness—namely, *ideation* and *volition*, with their associates, *memory* and *emotional sensibility*,—the agency and instrumentality of the hemispherical ganglia are involved; in other words, the organs of the perceptive consciousness, in the cerebrum, are in effective operation.

We have also seen there are valid reasons for the belief, and I have avowed my own conviction on the point, that the great internal convolutions—the *ourlet of Foville,* and essentially the basement convolutions of the cerebrum—are the central organs of the perceptive consciousness, the portals to intellectual action, where sensory impressions, the intuitions of the special senses, whether sights, sounds, tastes, smells, or feelings, become *idealized* and *registered*—that is, *perceived*, *remembered*, and *associated*; and where, too, the *ideation* of outward *individualities* is effected: for the senses are the inlets,—the media which connect us with the world without,—through them the perceptive consciousness is reached, and to their intuitions we owe our knowledge of the sensible qualities of external existences. In the perception proper, indeed, of mere outward objects, as in the intuitions of the special senses, man stands on the same platform with the other vertebrata, for the process in each and in all is the same. The lower animals, by virtue of their perceptive consciousness, not only perceive external objects, but remember actions and incidents associated with them. They shun danger after past experience, and act voluntarily. They have evidently an intuitive sense of time, space, form, and distance. And besides the animal propensities, they display social attachments, desires and aversions, angry passions and joyous feelings. But in them, it is obvious, *ideation* has reference, *instinctively*, either to the satisfaction of their appetites or to self-preservation. It cannot escape observation, that the *conservation* of the individual and the *multiplication* of the species are their dominating instincts; and that in the fulfilment of these, all the sagacity and laborious industry which they manifest are exerted. It may be, indeed, fairly inferred that the intuitions of the special senses, and their allied feelings, appetites, and instincts, form the chief and predominant part of the mental life of the inferior animals; but, at the same time, it must not be forgotten that these, too, constitute the inferior region of the true or conscious mind, and enter largely into the complicated web of human existence.

The instinctive attachment of the mother to her offspring, but limited in duration to the period of its helplessness, appears to be as great among some of the inferior tribes as it is among many of the human races. But this attachment, even among social animals, ceases with the period of infancy and helplessness ; and in their after life, those affections and endearing relations which are the charm of human life, have no existence. The attachment of the dog to his master is an enduring theme, but "it may be doubted whether we can find any instances of such feelings between animals themselves, excepting some cases of sexual unions. In general, they seem entirely destitute of sympathy with each other, indifferent in each other's sufferings or joys, and unmoved by the worst usage, or acutest pangs, of their fellows."* They are destitute of moral instincts.

The dog *knows* his master, and *remembers* scenes and actions in which they have been associated together. He may be said to have well-nigh all the rudiments of our perceptive knowledge—*ideation*, *emotion*, and *volition ;* but he holds them in an instinctive form. He recognises his master by certain characteristics; but disguise those, and you balk his instinct. He is deficient in *reflective* as opposed to *immediate apprehension*, and of him it has been aptly said, "*Though he knows the person, he does not know how he knows.*" In the expressive language of Burns,

"*Man is the god of the dog.*"

But man *perceives relations*, as well as external objects and their physical adjuncts. He notes events and circumstances in their *relations* to time and locality, or space. He is a *thinking* as well as a sensitive being, but every cogitative act necessarily implies an apprehension of the *relations* of the objects of *thought*. It involves a process of *comparison*, and the result is a *perception* of resemblance and difference. All knowledge, indeed, is a *relative* apprehension of things : and a *relation* cannot be rightly apprehended until we are equally familiar with both its terms, and until we can take our stand at either end and contemplate the other at will. And is it not this ability to shift the mental action, and to deal freely with the two sides of a *relation*, which constitutes the genuine mark of human intelligence, as distinguished from mere animal sagacity ?

Now, in the perceptive consciousness of man with the great central organs, where *ideation* is effected, there are not only associated the super-orbitar convolutions, through the instrumentality of which he acquires a knowledge of the physical adjuncts of natural objects, such as their size, form, colour, number, &c., but a still higher order of intellectual organs and faculties, by virtue

* Lawrence's Lectures on Physiology, Zoology, and the Natural History of Man.

of which he rises to the apprehension of the qualities and properties of external objects, and to a knowledge of their intimate structure and mutual relations, totally and altogether different from any intelligence that can take place in the case of the inferior animals.

In the emphatic language of Professor Sedgwick, "Man stands by himself, the despotic lord of the living world; not so great in organic strength as many of the despots that have gone before him in nature's chronicle, but raised far above them all, by a *higher development of brain*,—by a special instinct for combination,—by a prescience that tells him to act prospectively,—by a conscience that makes him amenable to law,—by conceptions that transcend the narrow limits of reason,—by hopes that have no full fruition here,—by an inborn capacity of rising from individual facts to the apprehension of general laws,—by a conception of a cause for all the phenomena of sense,—and by a consequent belief in a God of Nature."*

Still, however, the elements of all his knowledge—intellectual, moral, and religious—come through the perceptive consciousness; for they have their origin or source in intuitional or perceptive experience, in their respective cerebral organs, through the medium of the central organs of the perceptive consciousness, with which they are connected and associated. It it thus, through the perceptive consciousness, by the inlets of the special senses, that man gains his first glimpses of the *true*, the *beautiful*, and the *good*—of sublimity in nature, and of harmony in sound. For, as Mr. Morell has justly observed, "no one can doubt but that the creation around us has been formed according to the most perfect laws of form and beauty, or that the human mind is so constructed that the idea of beauty must, under the highest culture, correspond with the teachings of nature; so that the mere presentation of the beautiful *without*, is as well calculated to awaken the intuition of it *in the consciousness*, as our ordinary contact with natural objects awakens the perception of their physical qualities." †

Again, no sooner has the perceptive consciousness become developed, than man, long before he has attained to the utterance of articulate speech, is able intuitively to interpret the tones, gestures, and expressions of emotion, and becomes sympathetically affected by them. Nay, more, an intuitive apprehension of *right* and *wrong* is attached to certain actions, and evidently *precedes* in his mind any distinct comprehension of the language by which moral truths are conveyed. The blush

* Sedgwick's Discourse on the Studies of the University of Cambridge. Fifth edition. Preliminary Dissertation.

† Morell's Psychology.

upon the cheek, and the early sense of shame, come before there has been any trains of thought as to the consequences of crime or misconduct. In the expressive language of Lord Bacon—"The light of nature not only shines upon the human mind through the medium of the *rational faculty*, but, by an *internal instinct*, according to the law of conscience, which is a sparkle of the purity of man's first estate."

So, again, "closely connected with the *moral*, are the *religious* intuitions of the soul. These are developed more or less distinctly amongst the earliest of our human sentiments, in the form of *awe*, *veneration*, and *reverence*, which are inspired by objects of sublimity, grandeur, vastness, and mystery. In process of time, other elements—first the mental, then the moral—are joined to the primary intuition, until at length we reach the elevation of an intelligent, voluntary, and cheerful dependence upon an Infinite and All-perfect Being."*

And thus we see that the original sources or germs of the elements of all his knowledge lie within the region of immediate experience in the perceptive consciousness. All knowledge consists in the perception of truth, and "*truth in the agreement of the sign with the thing signified;*"† and hence it follows that the elements of knowledge are so many and as different as are the various fundamental truths, intellectual, moral, and religious, which they embody. But in all our psychological investigations we must never lose sight of the important fact, that the human mind, from its earliest existence, comprehends *implicitly*, and that, too, in the very nature of its existence, *everything* which its interior nature is calculated afterwards to develope. These germs, or essential elements, as constituent endowments, exist in every *mens sana*, *implicitly*, from its earliest existence, and they are one and all evolved *explicitly* through the perceptive or intuitive consciousness.

True it is, that by no training or culture can we create a new faculty, any more than we can invent a new law of nature, or give a new organ of sense. But, nevertheless, it is equally true that the germs (so to speak) of all our mental activities, intellectual, moral, and religious, *are present from the first*, and they are all evolved through the perceptive consciousness, with the collateral development of the vesicular matter of the cerebral organs, through which they are manifested throughout the totality of life; at first, in merely an instinctive and impulsive manner, and in the order and succession in which each specific form of mental activity is roused into prominent and effective operation, *by virtue of its reaction with the external world or*

* Morell's Psychology.

† Dr. Wollaston's Definition of Truth.

*nature.* To Mr. George Combe* belongs the honour of having first clearly demonstrated that the harmony which exists between the constitution of external nature and the mental constitution of man, is an all-pervading principle of creation, and a perfect and beautifully symmetrical system; and by bringing into one point of view the different constituent elements of the human constitution, and showing their relations to each other and to external nature, he has thrown a flood of light upon the phenomena of human life, and has indisputably established the fact, that the world, throughout its constitution, is framed in admirable adaptation to the faculties of man, as an intelligent, a moral, and a religious being.

We have seen that the mind, like the body, passes through its phases of development, and that at birth man is the mere creature of sensation and instinct; for in the earliest stage of his psychological progress, the *intelligence* is purely sensational, the *feelings* simply those of pleasure and pain, and the *impulses to action* innate and instinctive. The senses, indeed, come into play from the moment of birth; for as the human infant nestles in its mother's bosom, *smell* is its guiding *sense*, and it is through *taste* that it satisfies its first instinctive want or craving. But from this state of isolation and subjective feeling, in which the mind may be said to be virtually passive, it gradually rises to the higher phase of development which we are now considering, of intuitional or perceptive consciousness,—a state of increasing activity, in which a degree of mental attention is necessarily evoked, and where the idealized impressions are retained in the memory, as *representative ideas*, with a measure of tenacity commensurate with the attention bestowed. For no sooner does the perceptive consciousness begin to dawn, than in the child's mind the image of its mother becomes associated with its idealized impressions of smell and taste; so that at the sight of her its little heart bounds with pleasure, and its joyous emotions are as evident to all, as its loudly-proclaimed disappointment makes itself understood when denied the gratification which its instinctive and inherent feelings crave.

When we view man, indeed, in his threefold capacity of a social, a moral, and an intellectual being,—and, accordingly, as endowed with intellectual faculties, with propensities and affections individual and social, and with moral and religious feelings and sentiments,—we cannot avoid the conclusion that *ideation is the first step in his intellectual progress,* nor that he is admirably fitted in all his capacities for the natural world without, and for the social conditions in which his Creator has placed him.

* *Vide* The Constitution of Man, considered in relation to External Objects, by George Combe.

*Ideas are the pabula of thought*, and form equally a constituent element in the *composite nature* of our animal propensities, and of our emotional and moral feelings.

Ideation is as essential to the very existence of memory, as memory is to the operations of thought. For what, in reality, is memory, but the fact of *retained idealized impressions* in the mind? And without these retained idealizations, embodied in the memory as *representative ideas*, where are the materials of thought, and how are the processes of thought to be effected? True it is, that the agency of volition is alike essential to memory; for unless the attention of consciousness, by an act of volition, be arrested, or directed and fixed upon the object, the *idealized impression* may be so evanescent and transitory, that, "like the baseless fabric of a vision," it may leave not a trace behind it.

I have advanced the opinion, that the basement convolutions of the cerebrum—*the great internal*, as the portals of intellectual action, are the central organs of the perceptive consciousness where *ideation* is affected. And we have noted the fact, it is only in man that they exist in their highest state of development, and that their connexions with the primitive convolutions in the anterior, middle, and posterior lobes of the brain are so multitudinous; commensurate, in my opinion, with the psychological importance of their office, as the central organs through which the other perceptive organs, intellectual, moral, and religious, are reached and associated.

The development and relations of these basement convolutions are in accordance with the extent and range of the perceptive faculties of the animal, and may be fairly taken as a criterion or measure in fixing its place in the scale of being. The true difference between man and the inferior animals rests specifically and fundamentally on the *greater number* and *higher nature* of his psychological endowments. They have many cerebral organs in common; but there are others which he possesses, of which they are altogether destitute; and this constitutes the immutable distinction between him and them. We know that it is only the anterior lobes of the brain which exist in fishes, birds, and reptiles, and that the middle lobes do not make their appearance until we ascend to the mammalian class. And hence how limited in number are the cerebral organs and psychological endowments of these animals, when compared with those of man; and hence, too, the important induction, that the psychical faculties which they do possess and manifest are attributes of—for they must have their material organs, their local habitation and abode, in—the *anterior lobes* of the brain. Now, all these animals, besides their dominating instincts of

*self-preservation* and of *reproduction*, have, in common with man, the sensory ganglia and organs of the special senses, the internal or basement convolutions, though necessarily limited to the anterior lobes, some of the super-orbitar and other convolutions at the basis of the brain, with a rudimentary and varying degree of anterior development. But they are alike destitute of that frontal, towering, and expanded development, and of those exalted intellectual endowments which are *the sole prerogatives of man.* They all more or less manifest the essential phenomena of the perceptive consciousness, ideation and its associates, memory and volition, emotional impulses and feelings, and, amongst the highest of the order, some traces of ratiocination. The melodious notes of the sylvan songsters attest indisputably their possession of the *organ* of *tune,* in common with man; the varying strains of the mocking bird and the articulatory exhibitions of the parrot, are equally conclusive of the presence of the *imitative faculty,* while the building of their nests speaks for the *constructive instinct* of the tribe. They have all an intuitive perception of external individualities, and of some of their physical adjuncts. In the mere perception, indeed, of external objects by sight, and in that of some of their sensible qualities by touch, or feeling, smell, and taste, man, standing on the same platform, is inferior to some of the lower creation. He has neither the far-seeing eye of the eagle, nor scent-smell, of the dog; but in the apprehension of the intimate structure and chemical composition of substances, of their properties and mutual relations, as well as of the adjuncts of physical objects—as of space, form, size, weight, colour, number, and order—how immeasurably he rises above their level, by virtue of the greater development and higher endowment of the cerebral organs which they have in common in the anterior lobes, and of others in the same lobes, which he possesses, but of which they are altogether destitute. Thus the dog, looking at an open printed book, sees the book itself, as an external object, just as clearly and as plainly as his master does, but he has no apprehension, nor can he be made to apprehend the things therein signified by the printed symbols. To him they are dead letters without meaning. His intuitive perception of individualities—his knowing his master, and perceiving the book, and his power of distinguishing between persons and objects, confer upon him the capability of a narrow education; while in man, with his intellectual endowments, the ideation of individual existences "yields that insatiable curiosity, that restless thirst for universal knowledge, which exhausts the mineral, vegetable, and animal kingdoms, imbibes all the information this diversified globe can supply, and impels him to scale the heavens, and take note of the wonders of the starry infinite."*

So again, in reference to the adjuncts of external existences, the organs of form, size, weight, and colour, in association with the central organs of the perceptive consciousness, "enable the inferior animals to distinguish individuals, and to know familiar objects from strange, to preserve their own equilibrium, to take pleasure in each other's striped and spotted skins, or splendid plumage; but in man, these perceptive powers stimulate to new creations. The impulse of form, aided by still higher faculties, shapes the marble into beauty, and almost inspires it with life,—that of colour, under the guiding hand of genius, flashes its creations upon the canvas, and brings back to our admiring eyes, in all their living energy, from times long past, deeds of heroic adventure, or the hallowed displays of divine benevolence."†

So, too, in his perception of the changes in external objects, of the phenomena of action, of *events* and their adjuncts, *time* and *place*, how immeasurably is he raised above them,—comprehending in its fulness the revolutions of the earth itself, before history had a name, and the history of his own species in every portion of the globe. In fine, through the instrumentality of those exalted intellectual endowments peculiar to man, and which have their acknowledged seat in the anterior lobes, "adorning his brow like a diadem," the faculties of calculation, of order or arrangement, of comparison and causality, of ideality and wonder, he can number the stars, and with instruments furnished by the higher mathematics, can weigh and measure the planets, assign their courses and times, mark out the path and anticipate the coming of comets, calculate the distance of the most distant nebula, and only terminate his investigations in the inaccessible depths of infinitude. He arranges every object that comes within his cognizance, whether material or mental. He perceives resemblances and differences, abstracts and generalises, analyses and combines, compares and infers, and "ascends from nature up to nature's God." From *ideality*—the imaginative faculty, the vivifying soul of music, poetry,

* Memoirs of Dr. Spurzheim, by A. Carmichael, M.R.S.A.

† *Colour Blindness.*—At a *post-mortem* examination of Dr. Dalton, Mr. Bally (formerly assistant to Dr. Spurzheim) pointed out an *imperfect* or *deficient* development of the convolution of the anterior lobes, the site of the organ of colour. "Here, then," says Dr. Wilson, "according to the judgment of those present, there appeared a marked deficiency in that portion of the brain which phrenologists regard as the organ of colour, in the person of the most famous example of *colour-blindness;* and though he were not famous, his case would deserve record, as the solitary one where the brain itself was examined."

*Researches on Colour-Blindness,* by Dr. George Wilson, F.R.S.E., page 106. 1856.

and eloquence, refining, exalting, and dignifying every object susceptible of improvement—springs his sense of the *beautiful;* and from *wonder*, that of the *sublime.*

But reverting to the animal and moral nature of man, the fact is equally manifest, that ideas form a constituent element in the composite nature of the animal propensities, and of our emotional and moral feelings. Dr. Carpenter has clearly pointed out the distinction between propensity and instinct, and has shown that in the former, *ideation* is involved. "Instinct," says he, in his able analysis, "is an expression for a certain series of phenomena directed towards a given pnrpose, but not really involving any other physiological or psychological actions than sensations and respondent movements; whilst *propensity is a desire for gratification, involving an idea of the object.*"

Among the personal affections of the Ego, the *love* of *life* is paramount, and around it are marshalled and associated those instinctive and inherent activities, or animal propensities, subservient to the defence and conservation of existence.

The instinct of *self-preservation* is an universal instinct, and the very first that is roused into action. To it all the special senses are necessarily and of course subservient; but first and foremost are those of smell and taste. It is the sense of smell which attracts and guides the human infant to the mammary gland of its mother, to satisfy an *internal want or craving.* Hunger and thirst, as instinctive and internal cravings or feelings, are implanted by the Author of nature, to use the words of Prochaska, in accordance with the "*lex nostri conservatio;*" and, as subjective sensations, they have their immediate seat in the vesicular nervous tissue of the stomach and mouth.

But the *propensity for food*—and in hunger we have both *appetite* and *desire*—involves for its gratification both *sensorial* and *psychical* agency. We all know by experience how a savoury odour will cause the mouth to water; but is it not equally true that the very thought of it, the mere recollection or recalling of the idealized sensation, will produce the same effect? To ensure the gratification of the propensity, and to satisfy the desire for food, befitting means and modes are to be devised and adopted; and these as assuredly involve and necessitate the agency of *ideas*, and in their execution, in the adaptation of means to ends, the active exercise of intellectual faculties.

We have seen that, in subserviency to the instinct of self-preservation, the sense of smell is *primordial;* and it is interesting to follow up the cerebral connexions of the olfactory ganglia, and to note that the peduncles are not only in commissural connexion with the great centres of sensorial feeling, the *thalami optici*, and with the *ourlet of Foville,* where the sense is *idealized* and *registered*, but with those primitive and basilar convolutions of the cerebrum which surround the fissura Sylvii, and are coeval in point of existence with the fissure itself.

"Each ganglion of the olfactory nerves is connected with the hemispheres by a long, narrow *commissure*, lodged in a triangular-shaped grove, and passing backwards, till opposite the *fissura Sylvii*, where it splits into three divisions; the most external of these, distinctly medullary, runs down the *fissura Sylvii*, to be connected with the *anterior extremity* of the *middle lobe;* the internal is connected to the *posterior internal surface* of the under part of the *anterior lobe;* and the middle, which is the shortest, and, strictly speaking, no more than the internal portion of the external, is connected with the *posterior* edge of the *anterior* lobe."* And thus by the earliest and guiding sense to the instinct of self-preservation, from its cerebral connexions in the encephalon, are we not also guided and led in our psychological researches to the *allocation* or seat of the psychical organ in hemispheres of the *alimentative propensity* in man?

Closely associated and interwoven with the *love of life*, and besides those immediately subservient and required for the mere support and conservation of existence, there are other active and definite animal propensities common to man and the lower animals. There is the instinct of *self-defence*,—the *combative propensity*,† for the protection of life; and the *destructive*, to provide for its sustenance; there is that of *cunning* or *secretiveness*, to lie in wait for the prey, or to elude the pursuer; that of *fear* or *cautiousness*, to shrink back from the encounter; and that of *courage* or *firmness* to face it openly. There is the *propensity to hoard food* for future use, and the *constructive ability* to provide for it a storehouse.

* Solly On the Brain, p. 286, 2nd Edition.

Dr. Andrew Combe has recorded an interesting case in which the *love of life* was a ruling passion of a lady upwards of sixty years of age, and in whom there was found, at the *post-mortem* examination of the brain, "an enormous development of one of the convolutions at the base of the middle lobe, so striking as to arrest instant attention. The corresponding part of the skull," says Dr. Combe, "showed a deep and extensively-moulded cavity or lid, running longitudinally, with high and prominent sides, and presenting altogether an appearance much more striking than in any skull I ever saw. Whether it may have any connexion with the love of life, is a circumstance which may be determined by future observations."—*Phrenological Journal*, vol. ii.

† Mr. Combe was present at the *post-mortem* examination of an old gentleman, who had long been remarkable for the mildness of his disposition and the courtesy of his manners, until suddenly, about four years before his death, he became irritable and violent in his temper. From being kind, gentle, and civil to his servants, he became irritable, excitable, and passionate. "In the left posterior lobe of the brain a cavity was found, two inches in length, lined with a yellowish membrane, into which blood had been effused and afterwards absorbed. Its centre was on combativeness, but it extended also into adhesiveness, and a small portion into philoprogenitiveness. The corresponding portions of the brain on the opposite side were sound."—*Combe's System of Phrenology*, 5th Edition, 1853, p. 252.

Again, besides these, there are the higher instinctive activities —the love of self, or *self-esteem*—the love of others, or *benevolence*—and the love of approbation; but these, although not exclusively human, are only found among the higher order of social animals. Now, to be satisfied that these are *primitive*, *distinct*, and *inherent* animal activities, all involving *ideation*, and roused into activity and exercise through the perceptive consciousness, we have only to appeal to nature. And the allocation of the cerebral organs of these activities by Gall and Spurzheim in certain primitive convolutions of the cerebrum at the base, surrounding the fissura Sylvii, and at the sides and upon the hemispheres, is not without the support of pathological evidence. And it is, if I am not greatly mistaken, to *post-mortem* examinations of the brain, and to pathological investigation, more than to any other source, that we are to look, not for the discovery of normal functions, but for evidence in support or refutation of the dogmata advanced by craniological observers. Such is the course I have kept steadily in view, and pursued with great interest, and not without advantage, in my limited field of observation.*

Throughout the whole creation, next in importance to the *love of life*,—the *instinct of self-preservation*, is that of *generation*. These are the two dominating instincts in nature. Locating, as I do, with Serres, the sensory ganglion of the sexual instinct in the median lobe of the cerebellum, as the result of personal observation and pathological research, it is highly interesting and instructive, in consequence of the direct commissural connexions of this sensory ganglion with the centres of emotional feeling, and through them with those of intellectual action, to trace the development of the composite character of the amative propensity in man, and to note how the instinct of

* In a case of tubercle of the brain, in a child, where the deposit was upon the superfices of the hemispherical ganglia, the psychological phenomena were most significant, and the sole indication of the local seat of the disease. At the *post-mortem* examination of the brain, the tubercular deposit was found to be situated on that part of each of the hemispheres of the brain where Gall and Spurzheim have located the *organ of firmness*. For some time previous to his illness, the parents of the child had been forcibly struck with a change in the disposition of the child, which they had observed for some time to be gradually taking place. From being a happy, placid, and docile boy, he had become more and more petulant, self-willed, and obstinate, very determined to have whatever he set his mind upon, and not to be driven from his purpose; in a word, he had become a most obstinate and self-willed boy. So marked, indeed, was the change of disposition, that it had become a subject of serious consideration with them, whether it was to be attributed to some latent disease under which he might be labouring, or to mere infirmity of temper. But as he continued to eat, drink, and sleep well, and did not appear to be suffering from any bodily complaint, they contented themselves with endeavouring to correct by moral management and discipline, what they were inclined to consider rather an infirmity of the mind, than of the body. I brought the case under the notice of the Royal Medico-Chirurgical Society. It was read, June 14, 1842, and published in vol. xxv. of the Society's Transactions.

propagation—one of absolute necessity—becomes a principle in our moral constitution, connected and associated with all our moral responsibilities, whilst, "at the same time, it furnishes materials for the imagination, taste, and perception of beauty." But in man, with the amative propensity, is inseparably associated and interwoven that of the *love of offspring*, of the *family circle*, and of *home*, knitting together in the bonds of affection, husband and wife, brother and sister, friend and friend. For, in accordance with high behest—"*Be fruitful and multiply, and replenish the earth*," the social affections bind family to family, and nation to nation, in one bond of universal brotherhood. Man is a social being, and the *love of society* or *propensity to associate* is inherent and instinctive. But among the lower animals, as I have already observed, the attachment of the mother to her offspring, however great for the time, is limited to the period of its infancy and helplessness; between them, in after life no endearing relations are observed to subsist. Now, why is this? Clearly and obviously because they do not possess, but are actually destitute of, the organs and faculties which administer to such relations.

For, of the posterior lobes of the brain, among the inferior animals we do not meet with the least vestige until we ascend to the carnivorous group. In the fulness of their development, these lobes essentially belong to the family of man, and are the great centres and seat of the psychical organs of his social propensities and attachments, and of the human affections.* Among monkeys and other anthropomorphous animals, there is a considerable development of the posterior lobes; and these animals are especially distinguished for attachment to their young, and for their social propensities; but, in them, they do not cover and overlap the cerebellum, as in man. Their great elongation backwards, and full development in the human brain, have led Professor Retzius to divide the whole family of man into *dolichocephalæ* and *brachycephalæ* in proportion to their breadth; and this division is not without psychological import among the races of man. Closely allied with the social propensities and human affections are the emotional states, and in them ideation is equally

* Mr. Combe mentions the case of a gentleman who died in Edinburgh, in whose brain "there were found at the *post-mortem* examination twenty-seven abscesses, of which eleven were in the cerebellum, and ten or eleven in the posterior lobes. There was only one in the anterior lobe devoted to intellect, and one was situated in the organ of tune, on the left side. He had made his will two days before his death, and to his physicians, his mind seemed to be entire. His brother, however, assigned as the reason why he desired the brain to be examined, that he had observed, that before his death, the deceased had manifested an almost *total loss of affection* for his wife and children, to whom, when in health, he had been tenderly attached. The coincidence between the seat of the disease, and the decay of the domestic affections is striking."—*Combe's System of Phrenology. Ante cit.*, p. 243.

involved; for, alike in the composite nature of each and of all, there is present an *intellectual element*, as well as *sensorial feeling*. Emotional is essentially different from common sensibility. We cannot identify hopes and fears, joys and sorrows, with the simple elementary feelings of pleasure and pain. The emotional differs from the sensational consciousness: they are distinct mental states. Still, the simple, elementary emotional sensibilities and impulses, like the instinctive feelings, are strictly consensual, and have their seat in the sensory ganglia; and as automatic functions of independent nervous centres, they may be brought into play through purely sensational channels, without the agency of volition or thought. Thus, laughter—*the expression of joyous emotion*—when excited by titillation on the surface of the body, is simply and strictly a *consensuous act*, as much so as the smile that mantles on the infant's countenance from the effects of flatus or some internal excitation. But the true emotional feeling involves ideation; and such is Laughter, "holding both her sides," when provoked by the presence of ludicrous ideas in the mind. In the one case, the physical impulse upon the surface passes *upwards* to the thalami optici; in the other, the ludicrous ideas are transmitted *downwards* from the centre of intellectual action in the cerebrum to them; and alike in both the motor impulses are instantly evoked, and the expression of the joyous emotion elicited. These facts were strikingly exemplified in the young woman's case to which I have more than once alluded; for at the time when her mental faculties were completely benumbed or paralyzed, and the only avenues open to emotional sensibility were those of sight and touch, through either of these channels feelings of fear and alarm, of terror and fright, could be instantly excited, with convulsive shuddering. And again, when she had so far recovered the power of ideation and observation as to perceive that her lover was faithless and paying attention to another, her emotional sensibility received a shock in another direction. She was *wounded in her affections;* jealousy was aroused; and the catastrophe followed, which, fortunately for her, proved salutary.

And thus we see that the two great centres of emotional feeling in the encephalon—the thalami optici and corpora quadrigemina, placed midway between the cerebrum and the external organs of sense, may be played upon and roused into action through either, from below or from above; *upwards*, from the *outer world*, by the appropriate stimulus upon the nervous vesicular expansion of each of the external organs of sense; *downwards*, from the cerebrum, from the *inner* or *psychical world*, by the flow of our thoughts, and the workings of ideo-dynamical, emotional, and moral agencies in our cerebral organs.

But the elementary emotional feelings and motor impulses, excited into action by impressions from without, bear the same relation, in the absence of the psychical element, to the true emotions, which the instincts do to the propensities.* Ideation is the connecting link—intermediating between the two extremes of mental action, emotion, and volition—between the inherent elementary emotional sensibilities on the one hand, and the operations of thought and volitional power on the other. As an intermediating and connecting link between emotion and volition, it is sometimes in subordination to the one, and sometimes to the other.† This is a point needing no illustration ; but

* It is greatly to be regretted that *perversions* of the *emotional feelings* should have met with such little attention in pathological researches.

† "During the past year, an interesting *post-mortem* examination came under my observation. It was the case of a little girl, aged eight years, who died on the eighth day of the attack, from effusion at the base of the brain, with softening of the pons Varolii. The manner of her death was very characteristic of the local lesion ; but the point of present interest was her impulse and emotional character while living. It was the theme of remark, and a matter of common observation, to all who knew her. I have never met with a more impulsive, excitable, curious, old-fashioned, and shrewd little girl, in the course of my life. I have watched her progress from infancy. She had a large head, and fully-developed convolutions ; but the size of the thalami optici was such as to rivet my attention, from their unusual magnitude and healthy appearance. I hope others will bear the comparative development of the thalami in remembrance in all cases where the impulse to emotional excitement has been characteristically great. Attention to this point is important, since it is only from multiplied observations that a safe and sound induction can be made."—*Physiological Psychology*, p. 68.

A striking instance of the dominant power of emotional apprehension—the *sheer dread of bodily pain*, in upsetting the balance of the mind, in the case of an intelligent, but highly impulsive and excitable lady, came under my notice in the summer of 1848. From that time, until within eighteen months of her death, which took place at Hornsey in the autumn of last year, she was under my observation. I was not apprized of her death until after she had been interred, which I sincerely regret, for no *post-mortem* examination was made, and there was an interest attaching to her case, in my mind, which nothing short of a knowledge of the pathological condition of the brain could satisfy ; and besides which, it was her own often-expressed wish to myself, to have her head examined after her death.

She was the daughter of a man of some public and political notoriety in his day,[1] and was begotten and born in the midst of a stormy period of her father's life. Between the ages of her parents there was considerable disparity. Her father was many years the older of the two ; and during the period of utero-gestation, her mother was the subject of great alarms and troubles, and underwent much anxiety and mental agitation. I mention these circumstances, because I think, with Dr. Latham, that, "prior to diseases, their diagnosis, their history, and their treatment —prior to them and beyond them, there lies a large field for medical observation. It is not enough to begin with the beginning. There are things earlier than their beginning, which deserve to be known. The habits, the necessities, the misfortunes, the vices of men in society, contain materials for the inquiry and for the statistical, systematizing study of the physician, fuller, far fuller, of promise for good to mankind, than pathology itself."[2]

When first called upon to see this lady, she was some months advanced in preg-

[1] Samson Perry, of the *Morning Chronicle*.

[2] Dr. Latham, On Diseases of the Heart.

even among the lower animals, those which habitually associate with man, it cannot escape observation that an intuitive comprenancy with her second child, and I soon found that the parturient state was the source of great mental uneasiness and discomfort to her. In consequence of the bodily pain and suffering which she had undergone at her first accouchement, she was looking forward to the next with fear and apprehension. So deeply, indeed, was the recollection of the first impressed upon her mind, that no sooner was she sensible that she had again conceived, than she became despondent and full of fear; nor could she bear the sight of the gentleman who had attended her in her confinement. His presence, associated as it was in her mind with her former suffering, made her quite miserable. It was in vain that I attempted to reason away her fears; they seemed to increase upon her as gestation advanced. The fear of bodily pain in prospect, the physical pains of labour, marred her present comfort, and rendered her quite unequal to the discharge of the relative duties of life. It was a kind of *monomania* with her—the dominant and depressing feeling of her mind. I found that positive assertion had more effect than reasoning or persuasion with her, and after a consultation with her husband, I assured her that she should be rendered insensible to pain when her labour came on. She was comforted with the idea; but I was not so comfortable, for I felt that I had promised more than I could perform. Ether and chloroform, as anæsthetic agents, were then unknown. She requested me to give her a written assurance that I would do this, which was done without a moment's hesitation; and this promise, up to the time of her delivery, she wore in her bosom, inclosed in a little silken bag. It was to her a talismanic charm. Whenever the desponding fit came on, and fear oppressed her, she read my promise, and was comforted. In this way, she got through the period of gestation, scarcely a day having passed without its being read. As soon as she was taken in labour, I was immediately summoned. I took with me an opiate (Battley's sedative). I held up the bottle to her, saying, "Here is your dose, but I cannot give it to you yet; you must be in actual effective labour, otherwise it will stop, at least protract, the process." I left, giving strict injunctions to the nurse not to send for me again until she thought I was really wanted; feeling assured from former experience, and as the event proved, that her emotional fears would vanish as the labour advanced. She had a safe and easy time, and a quick recovery. All allusion to her former state of despondency was carefully avoided. She nursed her child, and quite regained, mentally and bodily, her usual health and strength. About twelve months afterwards, I had a morning visit from her; she was again *enceinte*,—smilingly she said, "I was very foolish last time, and now I am beginning to *fear* again; but I know I can be saved the pains of labour this time, and I come to ask whether you will give me chloroform." To this I readily assented, and with this assurance, and the prospect of immunity from pain before her, she went on more comfortably until her time was up. Then her labour came on so rapidly, that before I could reach the house, the child was born. A sever flooding followed; she was greatly exhausted, and had a very protracted recovery. She was weak for a long time, both in body and mind—depressed, and despondent.

Again she became pregnant, and during the period of gestation had a severe attack of toothache. The pain brought back all her fears and apprehensions; but, under the influence of chloroform, the decayed tooth was removed, and this gave her fresh courage. Hope revived, and, with occasional fits of depression and fear, she struggled on. In due time, and under the influence of chloroform, she was safely delivered; but she never recovered her former healthy tone of mind. She was excitable and irritable, easily put out of temper, and very despondent. With a view to the benefit of her health, she left London, and went to reside at Hornsey. Once more she became pregnant, and all her emotional fears and apprehensions returned, aggravated in degree, and of so alarming a character, that medical advice was sought for in her immediate neighbourhood, and eventually Dr. Ramsbotham was consulted. Interested in her case, he wrote to me, and we had some correspondence on the subject. Finding, or at least thinking, that her medical attendant had not had much experience in the administration of chloroform, nothing would satisfy her mind, but that she must be near me, that I might attend her in her accouchement. Accordingly, she came to lodgings in Norfolk-street, a month before her hension of his emotional nature is acquired, which enables them at once and without hesitation to recognise its manifestations.

confinement. I saw her daily. She was in a state of monomania. Fear, the dread of her approaching accouchement, seemed to be never absent from her mind. I introduced my friend Dr. Snow to her. He gave her the most positive assurance that as soon as ever labour had begun, she should be rendered perfectly insensible to pain. Still she was full of fears : he might be otherwise engaged when she required him,—I might be from home, and she was quite sure that the agony of the first pain would kill her. Her mind was quite unsettled—she could attend to nothing; morning, noon, and night, the fear and apprehension of the bodily pains of labour in her mind were uppermost.

Fortunately, when she was taken in labour I was at home, and Dr. Snow was quickly in attendance. She was rendered completely insensible, and she had no knowledge of her child's birth until after it had been washed and dressed, when it was presented to her by the nurse. She seemed pleased that her trouble was over, but at first she could scarcely believe the fact. For the first two or three days, everything seemed to be going on satisfactorily; she was composed and quiet, and it was vainly hoped she would regain a healthy tone of mind, as she had done before. The event proved otherwise, for soon the *fear* of death took possession of her. The slightest bodily pain, any griping of the bowels from the effects of aperient medicine, or spasm from flatulency, produced a paroxysm of despondency and fear.

She lost all self-control. She took no heed or interest in her child. At first, for a few times, she attempted to nurse it ; but the pain of suckling she either could not or would not endure. She said, the pain of nursing would be her death. It was in vain to attempt to reason with her on the groundless character of her fears and apprehensions. She did not appear to fear death in connexion with her own state in the world to come; but what she dreaded was, the *pain*, the *agony* of *the act* of dying. I had consultations on her case with Dr. Locock and Dr. H. Monro, and it was found necessary to have a female attendant from an asylum to be with her. At times she was violent, under excitement; but as she was easily controlled, private surveillance sufficed, and she was never removed from the care of her friends. From the connexion of her malady with the puerperal state, hopes were entertained, which were never realized, that ultimately she might regain a healthy tone of mind. She returned to her family at Hornsey, but neither mentally nor bodily was she ever again able to discharge the relative duties of life.

From this time I lost sight of her ; but she was an invalid for the remainder of her life—the victim of illusionary ills and despondent feelings. "For the last three months of her life," writes Mr. Hands, of Hornsey, her medical attendant, in a letter which I received from him after her death, "I had to sustain a sinking and enfeebled frame. She was exhausted to the last degree ; I never saw a frame so denuded of muscular and adipose substance. Life was sustained for several weeks on the smallest possible quantities of food. Her perceptions to the last hour of her existence were acute, and she often said she could not die, and seemed to think that the ordinary course of nature in her case would be reversed." But, "*Lex non pœna, perire;*" although with her it was the dread of the *pain* of the *agony* of dying which poisoned the cup of life. How interesting and how instructive in her case it would have been to have known the pathological condition of the thalami optici, where the association of bodily pain with emotional despondency, was so prominently though painfully exemplified !

I have at this time a lady under my care, with whom any emotional excitement is attended with the loss of the memory of words, and even of the names of common things. Her case is not without interest. She is about fifty years of age, the mother of a large family, and of an impulsive disposition. In June, 1855, the unexpected failure of the bank of Messrs. Strahan, Paul, and Co., in the Strand, where her husband usually had a very considerable balance of money, was suddenly told to her, and under circumstances which gave her nervous system a shock. Seeing her husband perplexed and annoyed, if not distressed, she bore up at the time, and suppressed the expression of her feelings ; but the next day, when walking out with her daughter, at the corner of a street they suddenly encountered a noble-

But in man's moral and religious attributes the inferior animals do not participate. These are exclusively *his sole prerogatives*, constituting an immutable distinction between him and the whole brute creation, but, equally with his social propensities and the true emotions, involving *ideation* in their manifestation and progressive development. The moral instincts of right and wrong, and the emotional feelings of awe and reverence, come before all teaching, and are aroused in their respective organs through the perceptive consciousness; but intellectual agency is needed for him to apprehend and understand the *basis upon which moral obligation rests*, and to constitute "*religion a reasonable service.*" The essence of his responsibility to God and his fellow-man has its foundation in the basis of his intellectual, moral, and religious nature.

Now, the transverse convolutions upon the upper surface of the cerebrum are *exclusively human*, for they are only to be found in the family of man; and the allocation, therefore, in these convolutions is no unreasonable procedure of the organs of man's carriage, as it was driving rapidly past them, and she instantly exclaimed, "*There goes the villain who has ruined and reduced us to beggary!*" She was then seized with a sudden giddiness, and all but fainted: immediately afterwards she began to talk quite incoherently, and it was not without difficulty that she was got home, when I was immediately sent for. It was some hours after the seizure before I was able to see her. She then said she "*was better, far better;*" but her mind was astray. She was evidently under fear and alarm, and did not understand what was said to her, or comprehend any question that I put to her, excepting the assurance that she was better. This assurance seemed to give her great satisfaction for the moment, but it was always followed by her saying, "Are you sure? Oh yes! I am better, much better,—but are you quite sure? Thank God!" &c. Her pulse was small, feeble, and irregular; the surface of body generally cold and clammy, and the forehead rather hot, but there was no complaint of pain in the head. An abiding sense of apprehension and depression of mind was a prominent symptom. On the following day, the pulse was more steady, regular, and had acquired more volume; but the face was rather flushed, and the forehead hot. An antiphlogistic mode of treatment, without depletion, was strictly pursued, under which she gradually improved. Her perceptive and thinking powers were soon regained. She knew where she was, and all the family about her, as well as myself; but the memory of words was for some time in abeyance. She could not recollect the name of any one, not even that of her own daughter, who was constantly with her—nor of the most familiar things in the house by which she was surrounded, as a chair, table, looking-glass, &c. She had a perfect recollection of past circumstances and events up to the time of her seizure,—understood anything that was said and done about her—felt deeply conscious of her own inability to recollect names and common words when talking—and at times such was her emotional sensibility in consequence, that she became annoyed and excited even to tears. In this case, it may be fairly inferred that the sudden shock to the nervous system in the first instance deranged the organic actions and normal co-relations of the emotional and intellectual centres. The giddiness and faintness consequent upon the *sudden outburst of emotional excitement* in the street, and indicative of disturbance in the balance of the circulation in the brain, was followed by delirium, and incoherent rambling *as a consequence*. The delirium was of short continuance, coherence of mind was soon regained, and the powers of thinking and reasoning were gradually though slowly restored; but there long remained, and there still exists up to this time, a manifest *dislocation* of the memory of words, to use an expressive term of Sir H. Holland, on the slightest emotional excitement or mental agitation.

those exalted moral and religious attributes or faculties which man *alone possesses*, and which raise him so high in the scale of being above the whole brute creation.

It was here that Gall and Spurzheim located the organs of the *moral sense*, or *conscience*, of *reverence* or *veneration*, of *awe* or *wonder*, and of *hope*, " which springs eternal in the human breast." And the allocation, founded as it was on an accordance of the external configuration of the cranium with observed mental manifestations, rests precisely on the same kind of evidence, on the same basis, as that which assigns to the high, towering, and expanded forehead *the organs of intellectual greatness*. I do not hesitate to avow my conviction, though my field of observation has been limited, that, so far as *outward* and *visible signs* can be taken as indices of the mental energy and power *within*, Gall, Spurzheim, and Combe have furnished the data and fixed the landmarks. It must be acknowledged that no one has studied the varying forms of the human cranium, with a view to their psychical significance, with so much care and attention, and on so extended a scale, as the illustrious Gall; for it was the labour of his life, and he was the founder of Physiological Phrenology.

The cranioscopic observations of subsequent observers, including Carus among the most recent, have all tended to establish the general positions of Gall. The fact, indeed, is indisputable, that the development of the cerebrum moulds and fashions, giving configuration, shape, and volume, with some well understood limitations, to its bony envelope—the skull, so that *craneoscopacy*, is, in truth, an appeal to observation and to nature.

(To be continued.)

